# Book Reviews

**Published:** 1900-10

**Authors:** 


### Book Reviews.

A Manual of Clinical Diagnosis by Microscopical and Chemical Methods. For Students, Hospital Physicians and Practitioners. By Charles E. Simon, M. D, Late Assistant Resident Physician Johns Hopkins Hospital, Baltimore. In one 8vo volume of 563 pages, with 136 engravings and 18 full-page colored plates. Philadelphia and New York: Lea Brothers & Co. 1900. [Price, $3.50.]

Nowhere in the field of medicine has advance been noted more than on the lines of clinical diagnosis. Greater accuracy here means better management, better treatment, greater professional success. Of late it has become necessary to add laboratory researches to the other means at command in order to insure the greatest possible accuracy, especially in doubtful cases. In other words, to exhaust all professional resources, the microscope, chemistry and bacteriology must be invoked, and the knowledge thus accumulated must be added to the information obtained by physical examination in order to make the clinical picture complete.

Simon was the first to publish a manual embracing laboratory methods of diagnosis and it was accepted with great favor at once. But little more than four years have elapsed since his book first appeared, and yet we now find ourselves confronted with a third edition demanding notice. The reviewer is first impressed with the fact that since the establishment of clinical laboratories in our medical colleges great changes have taken place in methods of teaching. The blood is now demanding primary attention in many diseases and is first considered in this book, to which about 100 pages are devoted. A number of most beautiful plates serve to give force and clearness to the text. The urine, always next in importance, has 225 pages assigned to it. The sputum, the gastric juice, and the feces are also very fully considered, while all the minor juices and secretions, such as those of the mouth, the vagina and other cavities and orifices of the body, are all duly discoursed upon in accordance with their importance.

In noticing the first edition we heartily commended it as an important aid not only to the specialist, but to the general practitioner. A somewhat detailed examination of this third edition serves to confirm our early judgment—an opinion which we here and now desire to accentuate. Such books are decided additions to the literature of medicine.

Report of the Commissioner of Education for the Year 1897–98. Volume I., containing Part I. Volume II., containing parts II. and III. Octavo, pp. 2640. Washington: Government Printing Office. 1899.

These two volumes contain fifty-five chapters of closely printed material, including tables, statistical matter and reports relating to education in different quarters of the globe. In the first volume, which contains Part I., there is a table showing the courses of study in medical schools throughout the United States, to which is appended an abstract of requirements for the practice of medicine in the various states and territories.

Volume II. contains Parts II. and III., in the former of which there is a chapter—No. XXX.— devoted to the consideration of medical inspection of schools. In several cities a system of daily inspection of schools by physicians is in vogue. This chapter is a compendium of information relating thereto and consists of a series of extracts from school and other official reports, from the writings and addresses of professional men and from other sources. A brief description of the system in Paris is also given.

In the first part, in Chapter XX., is considered education in Cuba, Porto Rico and the Philippines, while in the preliminary matter of Part I. an educational policy is presented for these islands.

The report is full of interesting material that may be studied with profit by educators, and is one of the best arranged documents of the kind that as been issued by the department of education.

---

Transactions of the Southern Surgical and Gynecological Association. Volume XII. Twelfth session, held at New Orleans, La., December 5, 6 and 7, 1899. Edited by W. E. B. Davis, M. D., Secretary. Philadelphia: Wm. J. Dornan, Printer. 1900.

This splendid volume of over 400 pages records the proceedings of this great association during its twelfth annual meeting, held at New Orleans, December 5-7, 1899. It contains thirty-six papers, most of which were discussed in extenso and some of which are admirably illustrated. In the association are grouped some of the most distinguished surgeons and gynecologists of the South, besides a few who reside North of the old imaginary line boundary, but now, happily, not dividing nor separating the two sections.

Dr. Joseph Taber Johnson, of Washington, who was the president, delivered an admirable address which betrayed intimate acquaintance with the profession and great experience in society work. The hospitality of New Orleans is famous and it entertained the association in accordance with its royal traditions. The scientific work of the association was fully equal to the high standard that has characterised the previous meetings of the organisation. Dr. A. M. Cartledge, of Louisville, was elected president and under his able direction the association is preparing for a meeting at Atlanta next month. The volume everywhere betrays careful editorial handiwork, a most important element in such a book.

TRANSACTIONS OF THE MEDICAL ASSOCIATION OF CENTRAL NEW YORK. Thirty-second annual meeting, held at Syracuse, N. Y., October, 1899. Wm. C. Krauss, M D., Editor. Volume VI. Buffalo Medical Journal Print. 1900.

This association again presents an admirable day's work in the eighty-five standard octavo pages which this volume contains. The publication of its transactions has stimulated the association to do most excellent work, and every member should feel an interest in continuing the plan that was put in operation so successfully some five or six years ago. The size of the page is admirably adapted for library purposes and should not be changed. An interesting meeting is in contemplation at Rochester, October 16, 1900, under the presidency of Dr. John Gerin, of Auburn.

